# INFLUENZA VACCINATION AND OLFACTION IN OLDER ADULTS: FINDINGS FROM HEALTH ABC

**DOI:** 10.1093/geroni/igae098.2473

**Published:** 2024-12-31

**Authors:** Yaqun Yuan, Keran Chamberlin, Chenxi Li, Zhehui Luo, Teresa Tian, Eleanor Simonsick, Honglei Chen

**Affiliations:** Michigan State University East Lansing, East Lansing, Michigan, United States; Michigan State University, United States; Michigan State University East Lansing, East Lansing, Michigan, United States; Michigan State University East Lansing, East Lansing, Michigan, United States; National Institute on Aging, Baltimore, Maryland, United States; National Institute on Aging, Baltimore City 0400 Baltimore City, Maryland, United States; Michigan State University, Michigan State University, Michigan, United States

## Abstract

Olfactory impairment is common in older adults and is likely associated with multiple adverse health outcomes. However, few risk or protective factors for olfactory impairment in older adults have been identified. We examined the cross-sectional association between flu vaccination and olfaction among 2507 participants of the Health, Aging and Body Composition study (aged 71-82 years, 51.7% women, and 37.4% Blacks). Olfaction was tested at the clinic visit Year 3 in 1999-2000, using the Brief Smell Identification Test. Olfaction was defined as good (test score 11-12), moderate (9-10), or poor (0-8). Participants were asked about receiving flu vaccines in the past year at each clinic visit from Years 1 to 3. We used multinomial logistic regression to estimate the odds ratio (OR) and 95% confidence intervals (CI) of flu vaccine reported at each clinic visit and olfaction tested at Year 3, adjusting for demographics, lifestyle, general health status, and flu occurrence within the past three months. At Year 3, compared with those not vaccinated, participants with flu vaccination were less likely to have moderate olfaction (OR=0.66, 95%CI, 0.51-0.85) and potentially fewer poor olfaction (OR=0.79, 95%CI, 0.60-1.02). The association appeared more evident in men and white participants. Similar findings, albeit slightly attenuated, were found in analyzing flu vaccination data reported at Year-1 and Year-2 clinical visits. Our study provides preliminary evidence that flu vaccination may be protective against olfactory loss in older adults, especially for men and the white race.

